# Supplementary material for: Residue Levels and Dietary Intake Risk Assessments of 139 Pesticides in Agricultural Produce Using the m-PFC Method Based on SBA-15-C18 with GC-MS/MS
Source: Molecules. 2023 Mar 8;28(6):2480. doi: 10.3390/molecules28062480 (PMC10058466; doi:10.3390/molecules28062480)
Supplement: Supplementary file 1 [file molecules-28-02480-s001.zip › molecules-2234476-supplementary.pdf]

Supplementary Materials

Residue Levels and Dietary Intake Risk Assessments of 139 Pesticides in Agricultural Produce Using the m-PFC Method Based on SBA-15-C_{18} with GC-MS/MS

Yue Wang [1,2,†], Tingjie Huang [2,†], Tao Zhang [2,†], Xiaoping Ma [2], GuangShuo, Zhou [2], Meiyao Chi [2], Xinjie Geng [2], Chunhao Yuan [1,*], Nan Zou [2,*]

[1] School of Chemistry and Pharmaceutical Engineering, Shandong First Medical University, Shandong Academy of Medical Sciences, Tai'an 271016, China

[2] Key Laboratory of Pesticide Toxicology & Application Technique, College of Plant Protection, Shandong Agricultural University, Tai'an 271018, China

*Correspondence: yuanchunhao2017@163.com (C.Y); zounan1226@163.com (N.Z.);

Tel: +086-0538-8242611 (N.Z.)

† These authors contributed equally to this work.

Table S1. Recoveries (%) (n = 5) and repeatability (RSD %) from samples spiked at 10 μg kg^{-1} of target pesticides in other eight types of fruits and vegetables.

Compounds	Watermelon		Melon		Asparagus		Lotus root		Strawberry		Cucumber		Crown daisy		Leaf lettuce	
	Recovery (%)	RSD (%)	Recovery (%)	RSD (%)	Recovery (%)	RSD (%)	Recovery (%)	RSD (%)	Recovery (%)	RSD (%)	Recovery (%)	RSD (%)	Recovery (%)	RSD (%)	Recovery (%)	RSD (%)
Acetochlor	98.7	4.3	94.4	8.6	97.3	10.4	84.8	8.5	106.0	5.8	93.1	4.0	95.4	8.3	89.0	6.8
Alachlor	97.2	8.2	89.7	5.0	100.8	3.3	78.9	9.6	103.0	10.2	95.3	6.9	97.4	6.3	96.2	9.7
Aldrin	99.8	3.3	91.9	3.6	95.0	7.9	92.2	6.2	101.8	6.0	88.8	12.9	87.3	7.5	84.0	5.0
Ametryn	89.6	7.1	75.8	9.6	95.0	7.0	89.0	10.9	95.3	7.2	93.2	10.1	76.3	9.1	79.1	8.8
Anilofos	86.0	7.4	82.0	10.5	98.8	14.2	92.5	5.9	94.5	7.0	86.5	12.5	84.2	6.2	73.4	6.5
Atrazine	96.4	7.1	77.1	13.1	92.6	6.0	89.2	13.0	104.3	8.6	92.6	5.1	101.6	3.4	91.3	6.3
Azoxystrobin	97.2	10.2	99.2	10.6	112.6	5.5	77.5	7.9	101.7	4.4	95.2	10.9	86.3	11.5	91.1	5.5
Benfuracarb	102.9	8.0	96.8	5.2	99.9	7.4	102.9	8.0	86.5	8.2	72.9	9.8	75.8	6.2	103.3	4.4
α-HCH	101.9	9.7	95.0	10.9	100.5	8.8	101.9	9.7	86.5	12.5	84.5	7.1	76.1	8.6	97.7	6.1
β- HCH	115.7	7.2	98.9	3.2	83.4	10.2	118.7	7.2	92.6	5.1	77.4	9.0	86.8	5.9	101.4	6.2
γ-HCH	98.4	6.2	86.1	8.3	117.2	13.0	75.8	6.2	95.2	10.9	104.8	7.4	72.5	7.6	90.3	5.5
δ- HCH	80.2	8.6	91.9	4.9	106.7	10.1	76.1	8.6	75.9	9.8	90.9	11.3	80.7	5.4	92.7	6.0
Bifenox	87.3	11.4	82.3	13.1	86.4	8.8	86.8	5.9	85.0	2.5	77.9	7.1	83.1	10.2	97.0	7.1
Bifenthrin	82.9	8.5	87.8	9.2	75.7	8.8	72.5	7.6	80.8	11.5	98.4	16.0	97.8	8.2	106.0	6.7
Boscalid	84.5	7.1	90.6	5.8	102.8	6.5	80.7	5.4	100.6	6.7	101.1	5.6	73.0	9.2	99.1	6.4
Bromobutide	79.4	9.0	88.3	8.4	94.9	9.4	83.1	10.2	87.9	8.3	85.4	14.8	82.4	13.5	102.2	5.1
Bromoxynil	104.8	7.4	99.6	6.6	95.3	13.5	97.8	8.2	102.8	4.2	88.2	5.2	99.0	7.0	99.7	5.5
Buprofezin	80.9	11.3	79.1	9.8	91.4	3.8	73.0	9.2	101.1	5.6	103.4	4.2	109.4	10.3	95.3	13.5
Butachlor	79.9	7.1	86.7	11.0	84.6	11.8	72.4	13.5	75.4	14.8	91.6	8.1	105.2	6.6	91.4	3.8

Butralin	97.7	15.5	80.8	11.5	83.8	8.5	99.0	7.0	88.2	5.2	98.7	3.4	105.5	7.2	84.6	11.8
Carbophenothion	112.0	9.8	100.6	6.7	88.2	7.1	100.9	4.6	81.7	5.7	83.0	8.5	96.6	4.7	83.8	8.5
Carboxin	112.0	4.6	87.9	8.3	85.3	5.0	95.7	4.6	84.8	7.1	104.0	13.1	91.3	7.3	88.2	7.1
Chlorfenapyr	109.7	5.5	102.8	4.2	100.9	4.6	88.8	8.6	78.7	5.9	97.1	6.9	105.1	4.8	85.3	5.0
Chlorfluazuron	96.6	10.5	105.7	8.5	95.7	4.6	108.7	7.2	85.7	7.7	93.6	5.6	107.8	8.6	100.9	4.6
Chlorpropham	110.9	5.2	87.6	6.4	88.8	8.6	97.7	8.6	75.9	7.5	99.2	6.2	108.8	6.0	95.7	4.6
Chlorpyrifos	106.3	4.0	97.9	5.0	108.7	7.2	82.4	8.5	80.5	16.5	78.2	13.6	107.9	4.4	88.8	8.6
Clodinafop-propargyl	100.4	6.7	94.6	4.6	107.8	8.6	95.7	10.5	110.9	5.2	87.6	6.4	111.4	7.4	108.7	7.2
Clomazone	106.2	2.4	81.5	11.8	108.8	6.0	100.1	5.6	106.3	4.0	97.9	5.0	85.3	5.0	83.1	10.2
Coumaphos	97.9	5.0	78.7	5.2	107.9	4.4	104.5	4.0	100.4	6.7	94.6	4.6	100.9	4.6	97.8	8.2
Cycloxydim	94.6	4.6	85.7	10.9	111.4	7.4	100.3	3.4	106.2	2.4	81.5	11.8	95.7	4.6	73.0	9.2
Cyfluthrin 1	81.5	11.8	75.9	3.2	115.3	13.2	101.2	6.3	75.0	10.5	72.9	11.2	88.8	8.6	79.4	13.5
Cyfluthrin 2	82.9	11.2	80.5	8.3	100.1	5.1	103.8	9.2	109.8	3.6	76.4	12.4	108.7	7.2	99.0	7.0
Cyfluthrin 3	81.4	12.4	74.8	4.9	100.4	3.3	110.0	10.2	95.1	5.9	97.9	5.5	97.7	8.6	90.9	7.4
Cyfluthrin 4	97.9	5.5	86.6	4.2	98.1	6.4	100.9	9.9	114.8	6.6	93.7	6.2	82.4	8.5	76.7	9.7
Cyhalothrin 1	93.7	6.2	92.3	8.5	91.7	3.8	92.5	4.4	74.4	12.4	74.8	14.2	95.7	10.5	97.1	6.5
Cyhalothrin 2	78.0	11.1	98.4	6.4	105.0	3.9	102.2	5.0	97.9	5.5	86.6	7.5	83.4	10.1	90.5	12.3
Cypermethrin 1	89.4	11.8	97.4	7.9	77.6	10.9	90.2	7.2	93.7	6.2	92.3	5.1	89.5	8.2	117.5	13.5
Cypermethrin 2	89.0	5.9	96.9	10.4	98.2	7.6	97.3	6.0	72.0	11.1	98.4	5.5	76.4	8.5	98.1	6.4
Cypermethrin 3	104.3	2.8	95.0	3.3	96.5	6.3	98.7	6.9	89.4	11.8	97.4	3.9	97.1	6.5	103.2	11.3
Cypermethrin 4	81.6	7.4	82.4	7.9	87.8	6.6	90.5	16.2	99.5	6.3	82.2	7.5	80.5	12.3	90.9	6.3
Cyproconazole	83.1	8.0	97.3	7.0	90.2	7.2	82.5	3.6	88.2	5.5	78.1	13.8	117.5	13.5	112.0	11.2
Cyprodinil	114.5	13.9	101.1	14.2	97.3	6.0	78.0	12.5	94.3	4.9	100.5	7.1	98.1	6.4	100.1	5.6
Deltamethrin	104.9	10.2	99.9	6.0	96.2	4.6	82.1	10.5	104.4	6.1	97.4	7.5	91.7	3.8	104.5	4

Diafenthiuron	107.3	6.2	109.2	5.5	95.3	5.5	75.0	8.6	96.1	11.2	90.8	10.6	105.0	3.9	100.3	3.4
Diazinon	78.1	8.3	103.5	7.4	95.7	6.0	89.0	10.2	78.1	13.8	90.7	8.0	97.6	10.9	101.2	6.3
Diclofop-methyl	85.0	12.6	95.2	8.8	81.3	11.0	93.3	9.3	100.5	7.1	103.5	7.9	98.2	7.6	103.8	9.2
Dieldrin	101.5	5.2	101.7	10.2	112.5	13.1	96.4	6.5	97.4	7.5	101.5	7.2	96.5	6.3	110.0	10.2
Diethofencarb	94.9	2.5	102.0	8.3	87.4	6.2	96.2	6.3	90.8	10.6	101.3	5.2	98.0	13.5	93.4	10.1
Difenzoquat	94.3	4.4	85.6	10.4	97.4	7.4	75.1	11.4	85.6	7.1	109.2	4.6	98.8	10.6	89.5	8.2
Diflufenican	100.2	10.6	91.3	7.5	88.9	8.2	98.8	5.8	91.3	5.9	95.6	7.8	106.6	10.3	76.4	8.5
α-endosulfan	80.2	6.3	83.3	3.9	93.3	9.3	90.3	6.6	83.3	8.2	76.7	10.2	101.3	5.8	88.1	5.9
β-endosulfan	91.7	5.2	80.4	6.8	91.4	6.2	93.9	9.4	80.4	5.9	98.3	5.5	118.6	10.5	95.7	9.1
Endrin	94.2	9.8	89.0	7.9	111.3	4.4	81.7	9.2	101.9	6.2	96.1	11.2	111.7	11.4	83.0	6.3
Ethofumesate	102.4	9.7	104.3	12.6	104.6	9.7	91.5	6.6	103.8	3.8	94.3	4.4	119.5	12.0	88.3	10.5
Etridiazole	83.5	10.2	81.6	10.4	90.2	9.4	96.1	6.8	107.2	7.3	100.2	10.6	109.6	5.0	90.6	6.2
Famoxadone	83.7	5.6	83.1	6.2	104.5	5.9	89.3	9.4	81.7	8.1	80.2	6.3	102.8	9.3	106.8	7.5
Fenarimol	82.8	8.8	104.5	8.3	109.0	6.2	92.6	4.4	109.0	11.0	91.7	5.2	95.8	5.9	96.2	4.6
Fenazaquin	89.8	7.1	104.9	12.6	108.6	6.2	106.4	7.4	107.2	4.6	94.2	9.8	88.3	10.5	98.2	7.6
Fenbuconazole	113.4	12.6	107.3	4.4	100.6	4.1	77.2	12.9	116.4	4.2	102.4	9.7	90.6	6.2	96.5	6.3
Fenitrothion	109.8	6.5	89.8	7.1	95.0	6.3	86.0	5.3	92.5	5.5	88.2	5.5	106.8	7.5	87.8	6.6
Fenobucarb	93.1	4.6	113.4	12.6	106.0	3.6	100.0	7.3	89.9	12.4	94.3	4.9	96.2	4.6	90.2	7.2
Fenoxaprop-P	98.1	11.4	96.2	6.8	81.3	3.4	93.4	9.8	86.3	9.5	104.4	6.1	95.3	5.5	97.3	6.0
Fenpropathrin	92.2	13.1	96.4	5.9	87.9	10.2	95.0	10.9	94.1	10.9	96.1	11.2	95.7	6.0	98.7	6.9
Fenthion	110.4	9.5	91.6	7.9	97.2	3.6	85.8	10.9	104.3	10.9	94.3	4.4	81.3	11.0	99.5	16.2
Fipronil	103.0	4.1	85.3	5.8	85.6	8.4	93.7	6.8	102.8	10.6	100.2	10.6	112.5	13.1	82.5	3.6
Fluazifop-P-butyl	113.9	8.2	85.0	7.3	104.9	7.8	88.2	10.3	104.3	2.8	95.0	3.3	87.4	6.2	80.0	12.5
Fludioxonil	101.3	8.6	102.5	8.8	91.7	5.2	87.3	6.3	81.6	7.4	82.4	7.9	97.4	7.4	82.1	10.5

Flufenoxuron	83.5	10.2	91.6	7.4	103.1	3.6	93.9	4.4	83.1	8.0	97.3	7.0	82.5	3.6	88.6	5.1
Flumioxazin	83.7	5.6	83.1	8.0	106.7	5.5	81.4	11.3	114.5	13.9	111.1	14.2	88.0	12.5	99.1	7.2
Fluorochloridone	82.8	8.8	114.5	13.9	100.3	7.3	82.1	8.4	104.9	10.2	99.9	6.0	82.1	10.5	99.1	8.8
Fluoroglycofen	89.8	7.1	104.9	10.2	98.9	3.8	72.0	6.5	107.3	6.2	109.2	5.5	75.0	8.6	106.1	11.4
Fluroxypyr	113.4	12.6	107.3	6.2	118.6	8.0	111.9	7.9	78.1	8.3	103.5	7.4	89.0	10.2	79.7	10.5
Flusilazole	96.2	6.8	78.1	8.3	93.4	9.8	96.4	7.8	75.0	12.6	95.2	8.8	79.1	10.5	95.8	9.4
Flutolanil	96.4	5.9	75.0	12.6	95.0	10.9	84.6	10.1	101.5	5.2	101.7	10.2	85.8	8.5	96.8	5.9
Fomesafen	92.0	8.2	87.3	5.3	85.8	10.9	106.8	7.2	98.1	11.4	96.2	6.8	79.7	7.1	95.6	6.2
Fonofos	116.0	10.4	103.7	11.1	93.7	6.8	87.2	6.5	92.2	13.1	96.4	5.9	92.7	6.2	109.3	6.0
Haloxyfop-P	116.7	7.5	119.1	15.4	88.2	10.3	94.9	8.2	110.4	9.5	91.6	7.9	87.1	9.4	104.7	3.3
Imazalil	102.5	3.9	72.5	9.4	100.3	8.2	86.9	10.2	103.0	4.1	85.3	5.8	93.2	7.1	93.5	8.5
Irgarol 1051	100.9	6.8	86.7	11.3	82.1	8.4	100.9	7.5	113.9	8.2	85.0	7.3	111.3	4.4	96.4	6.5
Isocarbophos	113.3	7.9	93.3	8.2	72.0	6.5	84.1	12.2	101.3	8.6	102.5	8.8	104.6	9.7	96.2	6.3
Isodrin	118.0	12.6	100.9	6.6	111.9	7.9	101.6	3.7	108.5	5.1	92.4	5.3	90.2	9.4	77.1	11.4
Isofenphos-methyl	88.5	10.4	93.6	5.9	75.5	11.5	72.9	7.0	106.5	7.0	90.7	6.6	104.5	5.9	98.8	5.8
Isoprocarb	99.3	7.5	85.7	6.7	100.0	4.0	97.9	7.1	92.0	8.2	87.3	5.3	109.0	6.2	90.3	6.6
Isoprothiolane	85.0	7.3	96.4	8.2	94.6	6.1	96.9	5.4	116.0	10.4	103.7	11.1	108.6	6.2	93.9	9.4
Kresoxim-methyl	102.5	8.8	75.7	8.6	97.7	7.6	97.5	8.1	102.5	8.8	75.7	5.5	100.6	4.4	81.7	9.2
Malathion	92.4	5.3	88.4	10.2	101.0	8.3	102.1	4.4	92.4	5.3	88.4	7.0	113.5	9.3	95.0	6.3
Mefenacet	90.7	6.6	84.6	5.6	101.4	4.4	77.6	5.2	90.7	6.6	84.6	8.4	101.2	4.7	106.0	3.6
Mepronil	87.3	5.3	76.4	8.8	87.8	6.5	82.8	11.2	87.3	5.3	76.4	15.6	81.7	9.2	96.2	10.2
Metazachlor	92.9	7.9	102.8	7.1	96.9	9.1	84.1	5.9	103.7	11.1	88.2	10.2	95.0	6.3	91.5	6.6
Methidathion	88.8	8.3	94.9	9.4	103.6	3.2	78.5	6.3	119.1	15.4	115.7	13.4	106.0	3.6	96.1	6.8
Metribuzin	93.5	5.2	80.9	5.6	101.6	10.2	89.0	6.6	72.5	9.4	99.0	4.9	81.3	3.4	99.3	9.4

Monocrotophos	93.0	11.0	82.5	7.5	101.7	4.9	94.4	8.6	86.7	11.3	78.1	9.9	87.9	10.2	92.6	4.4
Myclobutanil	92.9	5.7	99.8	5.5	96.9	5.4	95.0	3.2	93.3	8.2	90.0	8.0	97.2	3.6	106.4	7.4
Napropamide	90.6	8.6	90.4	5.9	97.5	8.1	95.0	5.7	88.2	10.2	90.3	5.6	85.6	8.4	77.2	12.9
Norflurazon	116.6	10.2	73.6	12.8	102.1	4.4	98.8	7.9	115.7	13.4	103.2	10.9	78.7	11.4	97.1	10.5
o,p’-DDD	87.4	9.4	92.2	6.9	78.8	6.2	92.6	4.7	99.0	4.9	105.4	6.2	89.7	12.5	96.7	7.4
o,p’-DDE	102.8	8.2	75.3	9.8	98.1	5.1	120.6	13.3	78.1	9.9	100.7	6.5	87.3	6.3	90.5	7.5
o,p’-DDT	99.3	8.0	76.8	10.6	98.7	4.3	95.1	5.5	90.0	8.0	100.3	5.3	93.9	4.4	99.3	8.5
Oxadiazon	91.0	5.3	88.8	10.8	91.9	3.6	82.7	8.2	99.5	6.3	84.8	6.3	81.4	11.3	95.4	4.2
Oxadixyl	111.9	9.2	87.8	11.8	75.8	9.6	99.8	3.3	117.9	14.8	93.9	6.3	82.1	8.4	96.4	7.8
Oxaziclomefone	102.1	6.7	84.8	9.4	82.0	10.5	89.6	7.1	98.4	6.3	111.9	10.2	72.0	6.5	84.6	10.1
Oxyfluorfen	108.5	10.9	98.1	5.3	87.1	13.1	86.0	7.4	98.4	4.6	103.1	12.0	111.9	7.9	106.8	7.2
p,p’-DDD	118.1	3.6	101.7	7.8	99.2	10.6	96.4	7.1	113.3	7.9	93.3	8.2	85.5	11.5	87.2	6.5
p,p’-DDE	86.1	5.0	118.9	9.5	89.6	12.4	97.2	10.2	118.0	12.6	100.9	6.6	100.0	4.0	94.9	8.2
p,p’-DDT	105.5	8.7	102.1	6.7	87.9	9.4	78.2	12.8	88.5	10.4	93.6	5.9	94.6	6.1	86.9	10.2
Parathion	107.1	10.1	88.0	14.2	107.5	6.3	96.5	5.8	99.3	7.5	85.7	6.7	102.1	6.7	84.8	9.4
Parathion-methyl	108.7	10.9	93.0	7.9	113.1	9.5	87.7	10.0	111.9	9.2	87.8	11.8	108.5	10.9	98.1	5.3
Penconzole	104.8	12.5	95.0	10.6	97.9	10.5	96.4	7.5	102.1	6.7	84.8	9.4	118.1	3.6	101.7	7.8
Pendimethalin	110.2	3.5	92.9	7.9	98.2	5.8	89.6	12.4	108.5	10.9	98.1	5.3	86.1	5.0	118.9	9.5
Permethrin 1	96.4	8.6	91.6	6.8	108.5	12.0	87.9	9.4	118.1	3.6	101.7	7.8	105.5	8.7	102.1	6.7
Permethrin 2	75.7	5.5	107.4	8.1	85.2	4.7	102.1	7.9	86.1	5.0	116.9	9.5	107.1	10.1	88.0	14.2
Phenthoate	88.4	7.0	103.6	4.0	92.1	6.5	88.9	5.0	105.5	8.7	102.1	6.7	108.7	10.9	93.0	7.9
Phorate	84.6	8.4	100.3	5.0	109.7	9.9	83.0	10.2	84.8	9.4	78.1	4.4	104.8	12.5	95.0	10.6
Phosalone	77.4	15.6	82.7	11.3	104.6	3.4	103.0	8.8	98.1	5.3	99.9	10.9	110.2	3.5	92.9	7.9
Phosmet	88.2	10.2	90.3	5.6	72.2	12.8	78.4	12.3	101.7	7.8	100.5	5.5	110.0	9.4	88.8	8.3

Pirimicarb	115.7	13.4	103.2	10.9	96.5	5.8	79.0	11.8	118.9	9.5	83.4	10.2	116.6	10.2	93.6	12.8
Pirimiphos-methyl	99.0	4.9	105.4	6.2	87.7	10.0	103.1	5.9	102.1	6.7	117.2	13.0	87.4	9.4	92.2	6.9
Pretilachlor	100.5	5.5	89.0	6.2	96.4	7.5	104.8	6.6	88.0	14.2	106.7	10.1	102.8	8.2	75.3	9.8
Probenazole	83.4	10.2	92.8	4.6	93.5	5.6	92.3	5.3	93.0	7.9	86.4	8.8	99.3	8.0	72.8	10.6
Procymidone	115.2	13.0	102.1	9.4	100.3	5.1	103.7	7.5	95.0	10.6	75.7	8.8	91.0	5.3	88.8	10.8
Profenofos	106.7	10.1	105.7	7.3	79.2	10.5	112.9	8.5	106.7	10.1	105.7	7.3	81.4	8.3	78.0	5.3
Prometryn	86.4	8.8	107.8	8.3	84.4	10.8	74.1	13.9	76.4	8.8	107.8	8.3	87.0	4.8	84.1	15.5
Propachlor	75.7	8.8	102.6	8.2	103.0	8.8	98.5	5.1	79.7	8.8	102.6	8.2	99.9	4.2	99.7	5.3
Propanil	102.8	6.5	107.7	7.1	72.4	12.3	105.4	11.4	102.8	6.5	107.7	7.1	94.8	6.5	102.7	10.3
Propargite	108.5	10.9	98.1	5.3	89.0	11.8	107.3	6.2	94.9	9.4	112.3	7.3	95.4	8.3	89.0	6.8
Propazine	118.1	3.6	101.7	7.8	105.8	8.5	92.6	4.7	80.9	5.6	99.1	3.8	83.1	10.2	97.0	7.1
Propiconazol 1	86.1	5.0	110.9	9.5	94.6	5.6	112.6	13.3	82.5	7.5	98.8	5.1	97.8	8.2	106.0	6.7
Propiconazol 2	105.5	8.7	102.1	6.7	84.8	8.5	95.1	5.5	99.8	5.5	105.7	6.2	73.0	9.2	99.1	6.4
Propyzamide	107.1	10.1	88.0	14.2	78.9	9.6	82.7	8.2	90.4	5.9	105.9	5.9	72.4	13.5	102.2	5.1
Pyraclonil	108.7	10.9	93.0	7.9	92.2	6.2	95.6	10.1	73.6	12.8	101.5	5.0	99.0	7.0	99.7	5.5
Pyriproxyfen	104.8	12.5	95.0	10.6	89.0	10.9	98.5	6.1	82.8	7.5	93.0	11.0	90.9	7.4	100.4	7.2
Quinalphos	110.2	3.5	92.9	7.9	87.1	13.1	77.2	12.2	106.4	7.1	92.9	5.7	76.7	9.7	106.0	7.9
Quizalofop-ethyl	110.0	9.4	88.8	8.3	99.2	10.6	95.0	10.9	108.9	8.5	90.6	8.6	97.1	6.5	103.2	11.3
Simetryn	93.0	11.0	82.5	7.5	79.6	12.4	98.9	3.2	86.8	13.1	116.6	10.2	90.5	12.3	90.9	6.3
Spirodiclofen	92.9	5.7	99.8	5.5	87.9	9.4	86.1	8.3	109.3	13.6	87.4	9.4	98.0	13.5	83.4	10.1
Sulfotep	90.6	8.6	90.4	5.9	102.1	7.9	76.2	8.2	104.2	7.0	102.8	8.2	98.8	10.6	89.5	8.2
Tebuconazole	116.6	10.2	83.6	12.8	88.9	5.0	97.9	5.2	103.0	10.1	99.3	8.0	106.6	10.3	96.4	8.5
Terbufos	87.4	9.4	92.2	6.9	83.0	10.2	80.4	10.2	102.1	6.6	91.0	5.3	101.3	5.8	88.1	5.9
Tetramethrin 1	102.8	8.2	75.3	9.8	101.9	9.7	99.9	6.0	95.3	8.2	81.4	8.3	118.6	10.5	95.7	9.1

Tetramethrin 2	99.3	8.0	72.8	10.6	118.7	7.2	93.1	4.4	102.8	8.2	75.3	9.8	111.7	11.4	83.0	6.3
Thifluzamide	91.0	5.3	88.8	10.8	98.4	6.2	86.1	12.5	99.3	8.0	72.8	10.6	119.5	12.0	88.3	10.5
Thiobencarb	82.5	7.5	98.8	5.1	90.7	7.9	79.2	13.0	91.0	5.3	88.8	10.8	109.6	5.0	90.6	6.2
Tolclofos-methyl	99.8	5.5	105.7	6.2	110.6	9.9	71.5	7.9	81.4	8.3	78.0	5.3	94.6	4.6	85.7	7.7
Tolfenpyrad	90.4	5.9	105.9	5.9	94.8	4.2	104.5	9.8	87.0	4.8	84.1	15.5	81.5	11.8	85.9	7.5
Triadimefon	83.6	12.8	101.5	5.0	98.9	7.5	81.4	9.8	99.9	4.2	99.7	5.3	92.9	11.2	90.5	16.5
Triadimenol	92.2	6.9	100.0	4.1	115.9	7.1	91.1	4.6	94.8	6.5	102.7	10.3	79.4	12.4	78.8	14.2
Triallate	75.3	9.8	105.1	7.1	100.6	2.3	99.7	5.3	109.0	9.2	89.0	6.8	97.9	5.5	86.6	7.5
Triazophos	72.8	10.6	104.5	7.0	80.4	10.2	102.7	10.3	98.2	7.5	76.2	9.7	93.7	6.2	92.3	5.1
Trifloxystrobin	95.3	8.2	81.4	8.3	99.9	6.0	89.0	6.8	84.0	4.3	106.0	12.2	79.0	11.1	98.4	5.5
Trifluralin	97.3	9.3	87.0	4.8	93.1	4.4	86.2	9.7	105.7	6.7	104.7	10.2	89.4	11.8	97.4	3.9
Vinclozolin	106.0	12.2	99.9	4.2	86.1	12.5	84.0	5.0	97.0	10.6	105.4	4.2	99.5	6.3	82.2	7.5
Zarilamid	104.7	10.2	94.8	6.5	90.4	3.8	71.1	8.8	85.8	4.4	100.5	9.6	103.5	7.1	98.2	10.3

Table S2. Recoveries (%) (n = 5) and repeatability (RSD %) from samples spiked at 10 μg kg^{-1}, LOD, LOQ, correlation equation, R^2 of target pesticides in leek samples.

Compounds	Spiked 10 μg kg^{-1}		LOD	LOQ	Correlation equation	R^2	Compounds	Spiked 10 μg kg^{-1}		LOD	LOQ	Correlation equation	R^2
	Recovery (%)	RSD (%)						Recovery (%)	RSD (%)				
Acetochlor	92.0	11.3	2.6	8.0	Y = 30594.4+2761.8*X	0.9998	Haloxyfop-P	76.0	12.9	0.3	1.0	Y = 61868.7+11167.8*X	0.9989
Alachlor	79.0	6.1	2.0	6.0	Y = 65135.6+8723.5*X	0.9996	Imazalil	107.7	9.1	1.7	5.0	Y = 95978.9+19574.9*X	0.9994
Aldrin	93.1	7.9	2.0	6.0	Y = 51909.7+7746.7*X	0.9998	Irgarol 1051	79.3	11.1	1.7	5.0	Y = 84994.2+12101.6*X	0.9993
Ametryn	90.2	10.3	3.0	9.0	Y = 30512.5+2345.4*X	1.0000	Isocarbophos	75.5	10.2	1.0	3.0	Y = 184502+26031.5*X	0.9997
Anilofos	81.2	6.0	0.3	1.0	Y = 13830.1+5966.6*X	0.9999	Isodrin	84.6	9.2	1.7	5.0	Y = 12814.2+4428.5*X	0.9999
Atrazine	94.8	16.3	2.6	8.0	Y = 23997.8+2883.5*X	0.9999	Isofenphos-methyl	79.6	3.4	0.2	0.5	Y = 599499.0+96476.3*X	0.9994
Azoxystrobin	79.7	11.7	2.0	6.0	Y = 66644.0+20629.0*X	0.9999	Isoprocarb	83.3	9.9	1.7	5.0	Y = -14729.9+13666.7*X	1.0000
Benfuracarb	74.9	15.2	3.3	10.0	Y = 27578.1+4390.7*X	0.9991	Isoprothiolane	85.2	16.2	1.7	5.0	Y = 41899.1+4712.3*X	0.9988
α-HCH	81.5	6.6	0.3	1.0	Y = 38458.7+10303.9*X	0.9999	Kresoxim-methyl	77.2	18.4	2.0	6.0	Y = 81510.9+15370.6*X	0.9993
β- HCH	92.5	11.1	1.7	5.0	Y = 34329.9+11185.6*X	0.9995	Malathion	84.9	7.9	1.7	5.0	Y = 64864.6+5496.8*X	0.9988
γ-HCH	77.2	12.1	2.6	8.0	Y = -18044.0+3102.4*X	0.9996	Mefenacet	84.7	3.1	1.7	5.0	Y = 278460.0+64675.4*X	0.9997

δ- HCH	82.1	6.6	0.5	1.0	Y = 75644.9+28675.7*X	0.9999	Mepronil	75.8	12.2	1.0	3.0	Y = 97989.8+21505.2*X	0.9994
Bifenox	85.9	7.2	3.3	10.0	Y = -14938.3+4490.1*X	0.9998	Metazachlor	79.4	11.8	0.7	2.0	Y = 49319.2+14334.6*X	0.9991
Bifenthrin	79.1	11.2	0.3	1.0	Y = 1113580.0+63831.2*X	0.9997	Methidathion	102.6	13.2	2.0	6.0	Y = 56677.4+5499.0*X	0.9999
Boscalid	81.4	6.5	0.03	0.1	Y = 2772.0+18780.5*X	1.0000	Metribuzin	83.3	2.3	1.0	3.0	Y = 23890.9+9988.6*X	0.9999
Bromobutide	110.5	15.5	2.0	6.0	Y = 18222.9+2646.6*X	0.9998	Monocrotophos	74.9	13.4	3.3	10.0	Y = 203592.0+10898.2*X	0.9997
Bromoxynil	79.2	18.4	3.3	10.0	Y = 43439.6+910.1*X	0.9995	Myclobutanil	92.4	10.6	1.0	3.0	Y = 167912.0+30794.0*X	0.9993
Buprofezin	82.2	14.5	2.0	6.0	Y = 69484.0+8285.5*X	0.9979	Napropamide	80.5	10.7	2.6	8.0	Y = 72788.1+6988.5*X	0.9999
Butachlor	73.8	15.2	1.7	5.0	Y = 55625.0+5913.1*X	0.9982	Norflurazon	86.9	3.0	1.7	5.0	Y = 40033.2+23723.2*X	1.0000
Butralin	96.9	11.2	0.3	1.0	Y = 25274.7+3120.2*X	0.9933	o,p'-DDD	74.8	8.4	0.3	1.0	Y = 638683.0+111267.0*X	0.9997
Carbophenothion	99.7	5.9	1.0	3.0	Y = -26540.8+11413.2*X	0.9999	o,p'-DDE	84.4	5.8	0.3	1.0	Y = 401085.0+79473.6*X	0.9994
Carboxin	86.8	8.1	1.7	5.0	Y = 115070.0+25631.6*X	0.9998	o,p'-DDT	88.4	5.2	1.7	5.0	Y = 553920.0+151316.0*X	0.9999
Chlorfenapyr	74.8	18.4	3.3	10.0	Y = 21503.5+2490.6*X	1.0000	Oxadiazon	81.1	15.9	1.0	3.0	Y = 72163.0+24212.8*X	1.0000
Chlorfluazuron	85.4	7.9	2.0	6.0	Y = 1423.0+4718.9*X	0.9990	Oxadixyl	86.1	12.7	0.2	0.5	Y = 36201.6+16692.3*X	0.9999
Chlorpropham	92.1	14.3	1.7	5.0	Y = 27146.9+6258.0*X	0.9997	Oxaziclomefone	105.4	8.8	1.0	3.0	Y = -36543.5+5563.0*X	0.9993
Chlorpyrifos	70.2	10.4	0.03	0.1	Y = 208307.0+14361.1*X	0.9992	Oxyfluorfen	111.8	15.2	0.3	1.0	Y = 31897.1+6376.2*X	0.9920
Clodinafop-propargyl	73.1	7.2	0.3	1.0	Y = 14117.7+11795.7*X	0.9999	p,p'-DDD	82.5	8.9	1.7	5.0	Y = 732503.0+170488.0*X	0.9997

Clomazone	93.8	8.1	0.3	1.0	Y = 28079.6+16651.2*X	0.9999	p,p'-DDE	77.2	5.7	0.2	0.5	Y = 193701.0+76411.1*X	0.9996
Coumaphos	77.4	7.8	1.7	5.0	Y = -51225.8+8571.5*X	0.9988	p,p'-DDT	86.4	8.6	2.0	6.0	Y = -36864.7+19358.2*X	0.9998
Cycloxydim	90.0	3.8	1.7	5.0	Y = -15595.6+7020.6*X	0.9997	Parathion	82.7	10.4	2.6	8.0	Y = 98832.4+8191.0*X	0.9994
Cyfluthrin 1	80.4	8.2	2.6	8.0	Y = 244468.0+2245.5*X	0.9965	Parathion-methyl	91.5	7.6	1.0	3.0	Y = -5332.3+6736.2*X	0.9999
Cyfluthrin 2	90.1	7.9	2.6	8.0	Y =123617.0+4135.3*X	0.9998	Penconzole	77.3	7.2	0.2	0.5	Y = 101101.0+16193.4*X	0.9989
Cyfluthrin 3	89.7	7.0	2.6	8.0	Y = 54008.9+1259.2*X	0.9996	Pendimethalin	86.4	12.1	0.3	1.0	Y = 155725.0+6416.9*X	0.9996
Cyfluthrin 4	105.7	9.1	2.6	8.0	Y = 33460.7+1391.4*X	0.9996	Permethrin 1	78.8	5.9	3.3	10.0	Y = 102042.0+7814.6*X	0.9995
Cyhalothrin 1	75.4	10.8	2.6	8.0	Y = 710252.0+36153.3*X	1.0000	Permethrin 2	82.6	7.8	3.3	10.0	Y = 84841.4+10914.2*X	0.9996
Cyhalothrin 2	82.9	6.0	2.6	8.0	Y = 285200.0+48864.3*X	1.0000	Phenthoate	89.0	6.5	0.2	0.5	Y = 41071.7+3645.8*X	0.9974
Cypermethrin 1	89.4	5.9	2.6	8.0	Y = 45121.4+4021.3*X	0.9993	Phorate	108.5	19.5	1.7	5.0	Y = 2885.1+1754.7*X	0.9997
Cypermethrin 2	82.6	8.3	2.6	8.0	Y = 34449.5+3876.7*X	0.9993	Phosalone	89.2	10.5	2.6	8.0	Y = 82350.3+12499.7*X	0.9999
Cypermethrin 3	104.7	7.8	2.6	8.0	Y = 84679.3+6553.9*X	0.9993	Phosmet	82.8	15.9	2.6	8.0	Y = 59888.7+1371.2*X	0.9991
Cypermethrin 4	83.8	5.5	2.6	8.0	Y = 131.30+7021.2*X	0.9999	Pirimicarb	79.3	17.9	1.7	5.0	Y = 20455.3+5878.0*X	0.9998
Cyproconazole	72.4	10.9	0.3	1.0	Y = 130374.0+29509.0*X	0.9992	Pirimiphos-methyl	121.1	10.1	0.3	1.0	Y = 29500.4+6260.2*X	0.9996
Cyprodinil	44.8	6.9	1.7	5.0	Y = 224573.0+26923.6*X	0.9992	Pretilachlor	72.3	14.0	1.0	3.0	Y = 120583.0+18066.0*X	0.9994
Deltamethrin	83.6	8.2	3.3	10.0	Y =9659.20+6547.1*X	0.9999	Probenazole	78.3	15.2	3.3	10.0	Y = 13960.1+2953.4*X	0.9992

Diafenthiuron	58.8	15.7	1.7	5.0	Y = -20194.3+6494.1*X	0.9987	Procymidone	100.2	9.6	1.0	3.0	Y = 15249.8+2802.8*X	0.9992
Diazinon	89.6	10.5	1.7	5.0	Y = 30641.6+4444.6*X	0.9996	Profenofos	84.7	10.6	1.7	5.0	Y = -22531.8+3965.0*X	0.9997
Diclofop-methyl	89.9	2.8	1.0	3.0	Y = 53626.0+38249.9*X	0.9999	Prometryn	91.0	6.4	1.0	3.0	Y = 30161.6+13591.6*X	0.9999
Dieldrin	70.6	11.3	1.7	5.0	Y = -1663.6+3551.1*X	0.9996	Propachlor	88.4	10.3	1.7	5.0	Y = 23912.2+3931.9*X	0.9996
Diethofencarb	73.7	5.8	1.7	5.0	Y = 69827.3+9532.6*X	0.9981	Propanil	91.0	10.0	2.6	8.0	Y = 14763.2+15189.3*X	0.9999
Difenzoquat	112.9	10.5	1.7	5.0	Y = 47200.9+31407.5*X	0.9999	Propargite	113.4	15.2	1.7	5.0	Y = -5952.8+2947.6*X	0.9960
Diflufenican	52.7	9.4	0.3	1.0	Y = 151759.0+27120.3*X	0.9998	Propazine	77.3	7.5	1.7	5.0	Y = 62574.5+16106.7*X	0.9998
α-endosulfan	108.4	14.7	1.0	3.0	Y = 11912.7+1929.9*X	0.9963	Propiconazol 1	98.9	13.7	1.7	5.0	Y = -15595.4+5702.3*X	0.9998
β-endosulfan	103.6	8.7	1.7	5.0	Y = -15720.9+1643.4*X	1.0000	Propiconazol 2	90.5	10.5	1.7	5.0	Y = 21801.6+5715.8*X	0.9997
Endrin	80.3	10.5	1.7	5.0	Y = 29154.9+2227.5*X	0.9995	Propyzamide	97.6	9.8	1.7	5.0	Y = 205561.0+33251.9*X	0.9995
Ethofumesate	94.6	15.8	3.3	10.0	Y = 43669.0+3781.0*X	1.0000	Pyraclonil	70.9	13.5	2.6	8.0	Y = 40088.0+4359.8*X	0.9993
Etridiazole	95.3	6.0	0.3	1.0	Y = 925.4+4084.1*X	1.0000	Pyriproxyfen	86.0	5.2	2.0	6.0	Y = 11545.1+16106.6*X	0.9997
Famoxadone	79.4	12.5	3.3	10.0	Y = 3996.0+7671.1*X	0.9997	Quinalphos	73.7	9.6	1.0	3.0	Y = 188965.0+33679.2*X	0.9996
Fenarimol	93.6	10.1	2.0	6.0	Y = 52196.9+22130.0*X	0.9997	Quizalofop-ethyl	78.3	3.4	1.0	3.0	Y = 49939.7+28026.1*X	0.9999
Fenazaquin	85.0	5.5	2.6	8.0	Y = 288047.0+89536.0*X	0.9999	Simetryn	99.1	11.6	1.0	3.0	Y = 41004.9+8863.0*X	0.9999
Fenbuconazole	96.9	2.6	1.0	3.0	Y = 85133.1+44963.0*X	0.9999	Spirodiclofen	75.6	8.2	0.2	0.5	Y = 19633.0+2336.3*X	0.9989

Fenitrothion	82.1	13.5	1.7	5.0	Y = -2468.5+2059.1*X	0.9989	Sulfotep	121.4	2.3	0.3	1.0	Y = 38090.6+8749.1*X	0.9996
Fenobucarb	80.8	5.3	1.7	5.0	Y = 21221.9+13196.2*X	1.0000	Tebuconazole	81.3	4.0	0.2	0.5	Y = 92475.8+19199.9*X	0.9999
Fenoxaprop-P	75.8	12.8	0.2	0.5	Y = 92198.3+47600.0*X	0.9999	Terbufos	90.4	7.2	0.3	1.0	Y = 9423.6+1866.4*X	0.9997
Fenpropathrin	126.0	12.8	3.3	10.0	Y = 1016750.0+45428.8*X	0.9999	Tetramethrin 1	92.9	8.8	3.3	10.0	Y = 29474.0+2683.4*X	0.9989
Fenthion	98.4	9.4	1.7	5.0	Y = -8312.9+1551.8*X	0.9968	Tetramethrin 2	90.5	10.3	3.3	10.0	Y = 140190.0+11217.3*X	0.9993
Fipronil	75.4	11.4	0.3	1.0	Y = 95066.1+13066.1*X	0.9991	Thifluzamide	87.3	11.2	1.0	3.0	Y = 222885.0+41155.8*X	0.9998
Fluazifop-P-butyl	90.7	9.0	0.03	0.1	Y = 32301.6+14917.4*X	1.0000	Thiobencarb	110.5	10.3	1.7	5.0	Y = 20733.6+2655.2*X	0.9990
Fludioxonil	87.5	11.6	2.6	8.0	Y = 119880.0+19960.1*X	0.9992	Tolclofos-methyl	100.8	9.4	1.0	3.0	Y = 52361.1+24470.2*X	0.9999
Flufenoxuron	80.6	16.8	1.7	5.0	Y = 2641.9+3029.5*X	0.9997	Tolfenpyrad	96.1	10.4	0.1	0.3	Y = 110311.0+23276.8*X	0.9997
Flumioxazin	88.0	5.1	2.0	6.0	Y = 17068.9+7247.0*X	1.0000	Triadimefon	74.4	10.9	1.0	3.0	Y = 235206.0+35802.5*X	0.9998
Fluorochloridone	92.1	10.5	2.0	6.0	Y = 43142.4+5959.9*X	0.9996	Triadimenol	89.3	9.4	0.03	0.1	Y = 960562.0+8463.4*X	0.9997
Fluoroglycofen	114.5	8.6	2.6	8.0	Y = -24423.1+1039.3*X	1.0000	Triallate	71.7	10.8	0.3	1.0	Y = 97996.0+8279.5*X	0.9953
Fluroxypyr	116.8	8.3	3.3	10.0	Y = 7116.9+1148.6*X	0.9988	Triazophos	74.3	12.6	1.0	3.0	Y = 24794.4+7136.9*X	0.9995
Flusilazole	82.8	8.7	0.7	2.0	Y = 139670.0+16040.1*X	0.9993	Trifloxystrobin	82.3	2.8	1.7	5.0	Y = 286577.0+46326.5*X	0.9996
Flutolanil	93.3	5.8	0.7	2.0	Y = 422115.0+167660.0*X	0.9999	Trifluralin	107.8	11.3	0.3	1.0	Y = 8444.6+6809.3*X	0.9998
Fomesafen	76.3	18.0	3.3	10.0	Y = 992.4+2356.7*X	0.9996	Vinclozolin	96.9	10.2	1.7	5.0	Y = 11614.2+3927.6*X	0.9994

Fonofos	82.7	6.1	3.3	10.0	Y = -9182.0+2671.5*X	1.0000	Zarilamid	77.0	14.6	3.3	10.0	Y = 81555.1+8413.4*X	1.0000

Table S3. Pesticides detected in fruits and vegetables from Shandong, China.

Pesticides	Mean value (mg kg^{-1})	Range (min-max)	No. of detected samples	No.of samples≥ MRL (%)	Pesticides	Mean value (mg kg^{-1})	Range (min-max)	No. of detected samples	No.of samples≥ MRL (%)
Acetochlor	0.278	<DL[a]-0.864	21(6.7%)	0(0.0%)	γ- HCH	<DL	<DL	0(0.0%)	0(0.0%)
Alachlor	0.013	<DL-0.013	1(0.3%)	0(0.0%)	δ- HCH	<DL	<DL	0(0.0%)	0(0.0%)
Aldrin	<DL	<DL	0(0.0%)	0(0.0%)	Imazalil	<DL	<DL	0(0.0%)	0(0.0%)
Ametryn	0.007	<DL-0.007	1(0.3%)	0(0.0%)	Isocarbophos	<DL	<DL	0(0.0%)	0(0.0%)
Anilofos	0.009	<DL-0.019	6(1.9%)	0(0.0%)	Isodrin	0.021	<DL-0.022	2(0.6%)	0(0.0%)
Atrazine	0.008	<DL-0.116	12(3.8%)	0(0.0%)	Isofenphos-methyl	<DL	<DL	0(0.0%)	0(0.0%)
Azoxystrobin	0.041	<DL-0.105	14(4.5%)	0(0.0%)	Isoprothiolane	<DL	<DL	0(0.0%)	0(0.0%)
Benfuracarb	<DL	<DL	0(0.0%)	0(0.0%)	Kresoxim-methyl	0.026	<DL-0.133	5(1.6%)	0(0.0%)
Cypermethrin	0.039	<DL-2.344	19(6.1%)	2(0.6%)	Malathion	<DL	<DL	0(0.0%)	0(0.0%)
Bifenox	<DL	<DL	0(0.0%)	0(0.0%)	Mefenacet	0.008	<DL-0.014	19(6.1%)	0(0.0%)
Bifenthrin	0.031	<DL-0.173	18(5.7%)	0(0.0%)	Mepronil	0.014	<DL-0.052	26(8.3%)	0(0.0%)
Boscalid	0.013	<DL-0.492	54(17.2%)	0(0.0%)	Metazachlor	<DL	<DL	0(0.0%)	0(0.0%)
Bromobutide	<DL	<DL	0(0.0%)	0(0.0%)	Methidathion	0.009	<DL-0.021	7(2.2%)	0(0.0%)

Bromoxynil	0.019	<DL-0.036	26(8.3%)	0(0.0%)	Metribuzin	<DL	<DL	0(0.0%)	0(0.0%)
Buprofezin	<DL	<DL	0(0.0%)	0(0.0%)	Monocrotophos	<DL	<DL	0(0.0%)	0(0.0%)
Butachlor	0.043	<DL-0.132	31(9.9%)	0(0.0%)	Myclobutanil	0.028	<DL-0.842	10(3.2%)	0(0.0%)
Butralin	0.007	<DL-0.007	1(0.3%)	0(0.0%)	Napropamide	<DL	<DL	0(0.0%)	0(0.0%)
Carbophenothion	<DL	<DL	0(0.0%)	0(0.0%)	Norflurazon	<DL	<DL	0(0.0%)	0(0.0%)
Carboxin	<DL	<DL	0(0.0%)	0(0.0%)	O,P-DDE	0.006	<DL-0.006	1(0.3%)	0(0.0%)
Chlorfenapyr	2.853	<DL-2.853	1(0.3%)	0(0.0%)	O,P'-DDT	0.016	<DL-0.016	1(0.3%)	0(0.0%)
Chlorfluazuron	<DL	<DL	0(0.0%)	0(0.0%)	Oxadiazon	<DL	<DL	0(0.0%)	0(0.0%)
Chlorpropham	0.010	<DL-0.01	1(0.3%)	0(0.0%)	Oxadixyl	<DL	<DL	0(0.0%)	0(0.0%)
chlorpyrifos	0.016	<DL-0.933	58(18.5%)	1(0.3%)	Oxaziclomefone	<DL	<DL	0(0.0%)	0(0.0%)
Clodinafop-propargyl	0.066	<DL-0.066	1(0.3%)	0(0.0%)	Oxyfluorfen	0.060	<DL-0.188	7(2.2%)	0(0.0%)
Clomazone	<DL	<DL	0(0.0%)	0(0.0%)	P,P'-DDD	<DL	<DL	0(0.0%)	0(0.0%)
Coumaphos	<DL	<DL	0(0.0%)	0(0.0%)	P,P'-DDT	0.042	<DL-0.042	1(0.3%)	0(0.0%)
Cybutryne	0.030	<DL-0.03	1(0.3%)	0(0.0%)	Parathion	<DL	<DL	0(0.0%)	0(0.0%)
Cycloxydim	<DL	<DL	0(0.0%)	0(0.0%)	Parathion-methyl	<DL	<DL	0(0.0%)	0(0.0%)
Cyfluthrin	0.026	<DL-1.389	29(9.2%)	0(0.0%)	Penconazole	<DL	<DL	0(0.0%)	0(0.0%)

Cyhalothrin	0.014	<DL-0.079	16(5.1%)	0(0.0%)	Pendimethalin	0.023	<DL-0.041	25(8.0%)	0(0.0%)
Cyproconazole	<DL	<DL	0(0.0%)	0(0.0%)	Permethrin	<DL	<DL	0(0.0%)	0(0.0%)
Cyprodinil	0.040	<DL-0.04	1(0.3%)	0(0.0%)	Phenthoate	0.014	<DL-0.014	1(0.3%)	0(0.0%)
Deltamethrin	0.015	<DL-0.081	13(4.1%)	0(0.0%)	Phorate	<DL	<DL	0(0.0%)	0(0.0%)
Diafenthiuron	<DL	<DL	0(0.0%)	0(0.0%)	Phosalone	<DL	<DL	0(0.0%)	0(0.0%)
Diazinon	<DL	<DL	0(0.0%)	0(0.0%)	Phosmet	<DL	<DL	0(0.0%)	0(0.0%)
Diclofop-methyl	0.018	<DL-0.018	1(0.3%)	0(0.0%)	Pirimicarb	0.006	<DL-0.006	19(6.1%)	0(0.0%)
Dieldrin	<DL	<DL	0(0.0%)	0(0.0%)	Pirimiphos-methyl	<DL	<DL	0(0.0%)	0(0.0%)
Diethofencarb	0.102	<DL-2.162	18(5.7%)	0(0.0%)	Pretilachlor	<DL	<DL	0(0.0%)	0(0.0%)
Difenzoquat	0.196	<DL-0.196	1(0.3%)	0(0.0%)	Probenazole	0.009	<DL-2.079	42(13.4%)	0(0.0%)
Diflufenican	<DL	<DL	0(0.0%)	0(0.0%)	Procymidone	0.042	<DL-11.27	64(20.4%)	4(1.3%)
α-endosulfan	<DL	<DL	0(0.0%)	0(0.0%)	Profenofos	0.075	<DL-0.114	14(4.5%)	0(0.0%)
β-endosulfan	<DL	<DL	0(0.0%)	0(0.0%)	Prometryn	0.006	<DL-0.018	8(2.5%)	0(0.0%)
Endrin	0.007	<DL-0.008	2(0.6%)	0(0.0%)	Propachlor	0.113	<DL-0.118	2(0.6%)	0(0.0%)
Ethofumesate	<DL	<DL	0(0.0%)	0(0.0%)	Propargite	0.006	<DL-0.006	1(0.3%)	0(0.0%)
Etridiazole	0.012	<DL-0.02	8(2.5%)	0(0.0%)	Propazine	<DL	<DL	0(0.0%)	0(0.0%)

Famoxadone	0.044	<DL-0.072	21(6.7%)	0(0.0%)	Propiconazole	0.049	<DL-0.118	10(3.2%)	0(0.0%)
Fenarimol	0.010	<DL-0.014	4(1.3%)	0(0.0%)	Propyzamide	<DL	<DL	0(0.0%)	0(0.0%)
Fenazaquin	0.000	<DL	0(0.0%)	0(0.0%)	Pyraclonil	<DL	<DL	0(0.0%)	0(0.0%)
Fenbuconazole	0.017	<DL-0.027	6(1.9%)	0(0.0%)	Pyriproxyfen	0.028	<DL-0.071	36(11.5%)	0(0.0%)
Fenitrothion	<DL	<DL	0(0.0%)	0(0.0%)	Quinalphos	0.010	<DL-0.012	2(0.6%)	0(0.0%)
Fenobucarb	0.050	<DL-0.085	4(1.3%)	0(0.0%)	Quizalofop	0.040	<DL-0.04	1(0.3%)	0(0.0%)
Fenoxaprop-P-ethyl	<DL	<DL	0(0.0%)	0(0.0%)	Simetryn	<DL	<DL	0(0.0%)	0(0.0%)
Fenpropathrin	0.054	<DL-0.076	27(8.6%)	0(0.0%)	Spirodiclofen	0.502	<DL-0.957	5(1.6%)	0(0.0%)
Fipronil	0.005	<DL-0.005	1(0.3%)	0(0.0%)	Sulfotep	<DL	<DL	0(0.0%)	0(0.0%)
Fenthion	0.007	<DL-0.007	1(0.3%)	0(0.0%)	Tebuconazole	0.011	<DL-1.599	69(22.0%)	0(0.0%)
Fenvalerate	<DL	<DL	0(0.0%)	0(0.0%)	Terbufos	<DL	<DL	0(0.0%)	0(0.0%)
Fluazifop-P-butyl	0.529	<DL-6.308	15(4.8%)	0(0.0%)	Tetramethrin	<DL	<DL	0(0.0%)	0(0.0%)
Fludioxonil	<DL	<DL	0(0.0%)	0(0.0%)	Thifluzamide	<DL	<DL	0(0.0%)	0(0.0%)
Flufenoxuron	<DL	<DL	0(0.0%)	0(0.0%)	Thiobencarb	<DL	<DL	0(0.0%)	0(0.0%)
Flumioxazin	<DL	<DL	0(0.0%)	0(0.0%)	Tolclofos-methyl	<DL	<DL	0(0.0%)	0(0.0%)
Fluorochloridone	0.177	<DL-1.781	10(3.2%)	0(0.0%)	Tolfenpyrad	0.006	<DL-0.006	1(0.3%)	0(0.0%)

Fluoroglycofen	<DL	<DL	0(0.0%)	0(0.0%)	Triadimefon	0.028	<DL-0.079	6(1.9%)	0(0.0%)
Fluroxypyr	0.007	<DL-0.057	6(1.9%)	0(0.0%)	Triadimenol	0.101	<DL-0.510	51(16.2%)	2(0.6%)
Flusilazole	0.009	<DL-0.012	4(1.3%)	0(0.0%)	Triallate	0.006	<DL-0.006	1(0.3%)	0(0.0%)
Flutolanil	0.006	<DL-0.006	1(0.3%)	0(0.0%)	Triazophos	<DL	<DL	0(0.0%)	0(0.0%)
Fomesafen	<DL	<DL	0(0.0%)	0(0.0%)	Trifloxystrobin	0.020	<DL-0.119	10(3.2%)	0(0.0%)
Fonofos	<DL	<DL	0(0.0%)	0(0.0%)	Trifluralin	0.038	<DL-0.077	8(2.5%)	0(0.0%)
Haloxyfop-P-methyl	<DL	<DL	0(0.0%)	0(0.0%)	Vinclozolin	<DL	<DL	0(0.0%)	0(0.0%)
α- HCH	<DL	<DL	0(0.0%)	0(0.0%)	Zarilamid	0.018	<DL-0.018	1(0.3%)	0(0.0%)
β- HCH	<DL	<DL	0(0.0%)	0(0.0%)					

[a] Detection limit

Table S4. MRM Transitions and Other GC-MS/MS Parameters for the Compounds.

Pesticides	RT(min)	Quantification transition (Collision energy (eV))	Confirmation transition (Collision energy (eV))
Acetochlor	16.32	146.1>117.1(20)	223.1>146.1(20)
Alachlor	22.22	188.1>160.1(10)	161.1>146.1(12)
Aldrin	17.56	263.0>192.9(32)	263.0>227.9(26)
Ametryn	16.14	227.1>212.1(15)	227.1>170.1(15)
Anilofos	16.35	226.0>184.0(15)	226.0>157.0(15)
Atrazine	12.18	215.1>200.1(10)	200.1>104.1(20)
Azoxystrobin	34.17	344.1>329.1(20)	388.1>345.1(15)
Benfuracarb	30.01	190.1>144.1(10)	164.1>149.1(10)
α-BHC	11.10	180.9>144.9(15)	182.9>146.9(15)
β- BHC	12.31	180.9>144.9(15)	182.9>146.9(15)
γ-BHC	13.66	180.9>144.9(15)	182.9>146.9(15)
δ- BHC	12.49	180.9>144.9(15)	182.9>146.9(15)
Bifenox	28.54	341.0>310.0(12)	311.0>279.0(15)
Bifenthrin	28.19	181.1>166.1(22)	165.1>139.0(25)
Boscalid	31.51	342.0>140.0(15)	344.0>142.0(15)
Bromobutide	15.27	232.1>176.1(10)	232.1>114.0 (10)
Bromoxynil	26.54	126.9>57.0(8)	126.9>67.0 (8)
Buprofezin	23.87	249.1>193.1(10)	172.1>57.0(10)
Butachlor	22.22	237.1>160.1(10)	176.1>146.1(10)
Butralin	19.14	266.1>190.1(15)	266.2>220.1(15)
Carbophenothion	26.13	342.0>157.0(10)	342.0>296.0(5)
Carboxin	23.65	235.1>143.0(15)	143.0>87.0(15)
Chlorfenapyr	24.69	247.0>246.6(5)	247.0>226.8(20)
Chlorfluazuron	22.61	321.0>303.6(25)	323.0>306.1(30)
Chlorpropham	10.07	213.1>127.0(15)	127.0>65.0(25)
Chlorpyrifos	18.20	197.0>169.0(15)	313.9>258.0(15)

Clodinafop-propargyl	26.71	349.1>266.0(15)	349.1>238.0(15)
Clomazone	12.25	125.0>89.0 (5)	204.1>107.0(15)
Coumaphos	30.66	226.0>163.0(20)	362.0>334.0(19)
Cycloxydim	15.29	198.1>81.6(15)	198.1>107.4(10)
Cyfluthrin 1	31.42	165.0>91.0(15)	163.0>91.0(12)
Cyfluthrin 2	31.54	165.0>91.0(15)	163.0>91.0(12)
Cyfluthrin 3	31.63	165.0>91.0(15)	163.0>91.0(12)
Cyfluthrin 4	31.67	165.0>91.0(15)	163.0>91.0(12)
Cyhalothrin 1	28.19	181.0>152.0(23)	208.1>181.0(10)
Cyhalothrin 2	28.32	181.0>152.0(23)	208.1>181.0(10)
Cypermethrin 1	32.59	181.0>152.0(25)	163.0>127.0(10)
Cypermethrin 2	32.88	181.0>152.0(25)	163.0>127.0(10)
Cypermethrin 3	33.37	181.0>152.0(25)	163.0>127.0(10)
Cypermethrin 4	33.70	181.0>152.0(25)	163.0>127.0(10)
Cyproconazole	24.31	222.1>125.1(20)	224.1>127.1(20)
Cyprodinil	19.59	224.1>208.1(20)	225.1>210.1(18)
Deltamethrin	34.37	181.0>152.0(20)	253.0>198.0(30)
Diafenthiuron	25.06	311.2>296.2(15)	254.15>220.1(15)
Diazinon	13.43	179.1>137.1(15)	137.1>84.0(10)
Diclofop-methyl	27.11	340.0>252.8(10)	253.0>161.5(10)
Dieldrin	23.06	277.0>240.9(10)	276.9>206.9(20)
Diethofencarb	18.08	267.2>225.1(8)	225.1>125.1(15)
Difenzoquat	23.61	234.0>232.8(15)	234.0>129.6(15)
Diflufenican	27.25	266.1>246.1(10)	394.1>266.1(10)
α-endosulfan	21.63	240.9>205.9(20)	271.9>236.9(18)
β-endosulfan	23.07	240.9>205.9(20)	271.9>236.9(18)
Endrin	24.13	280.9>244.9(12)	280.9>244.9(5)
Ethofumesate	17.27	207.1>161.1(10)	286.1>207.1(12)
Etridiazole	6.81	210.9>140.0(15)	213.0>184.9(10)

Famoxadone	34.27	224.1>196.1(10)	330.1>224.1(10)
Fenarimol	29.59	139.0>111.0(15)	219.0>107.0(15)
Fenazaquin	28.44	145.1>117.1(15)	160.1>117.1(20)
Fenbuconazole	31.08	198.1>129.0(10)	129.0>102.0(15)
Fenitrothion	17.08	277.0>260.0 (10)	277.0>109.0(20)
Fenobucarb	9.20	150.1>121.1(10)	121.1>77.1(15)
Fenoxaprop-P	30.06	361.0>288.0 (10)	288.0>260.0(10)
Fenpropathrin	28.32	181.1>152.1(23)	265.2>210.1(15)
Fenthion	18.10	278.0>108.6(25)	278.0>168.5(25)
Fipronil	20.74	367.0>213.0(25)	213.0>178.0(16)
Fluazifop-P-butyl	24.76	383.1>282.1(15)	383.1>254.1(20)
Fludioxonil	23.59	248.1>154.0(20)	248.1>182.0(15)
Flufenoxuron	14.28	305.0>125.9(20)	307.0>125.8(20)
Flumioxazin	32.63	287.1>259.1(15)	354.1>326.1(10)
Fluorochloridone	18.92	187.0>159.0(10)	172.0>144.9(15)
Fluoroglycofen	29.85	223.0>131.7(15)	344.0>178.8(15)
Fluroxypyr	27.17	181.0>133.8(30)	209.0>178.5(25)
Flusilazole	23.94	233.1>165.1(20)	233.1>152.1(20)
Flutolanil	22.96	173.1>145.1(15)	281.1>173.1(15)
Fomesafen	18.93	311.0>173.6(15)	313.0>173.7(15)
Fonofos	12.90	137.0>109.0(10)	246.0>137.0(10)
Haloxyfop-P	21.78	375.0>288.0(25)	316.0>91.0(25)
Imazalil	13.00	175.0>147.0(16)	173.1>145.0(20)
Irgarol 1051	20.48	182.0>108.7(10)	238.0>181.6(10)
Isocarbophos	18.57	136.0>108.0(15)	230.0>212.0(10)
Isodrin	19.05	263.0>192.9(30)	263.0>190.9(30)
Isofenphos-methyl	19.64	199.1>121.0(15)	241.1>199.1(10)
Isoprothiolane	23.01	290.1>118.0 (15)	290.1>204.1(15)
Kresoxim-methyl	24.25	206.1>131.1(15)	131.1>116.1(20)

Malathion	17.73	173.01>99.0(10)	127.0>99.0(10)
Mefenacet	29.27	192.0>136.0(15)	193.1>137.0(15)
Mepronil	25.80	269.1>119.1(10)	269.2>210.1(10)
Metazachlor	19.76	209.1>132.1(12)	133.1>117.0(20)
Methidathion	21.24	145.0>85.0(10)	145.0>58.0(15)
Metribuzin	15.29	198.1>82.0(20)	198.1>89.0(16)
Monocrotophos	28.23	192.1>164.0(12)	192.1>127.0(10)
Myclobutanil	23.76	179.1>125.1(15)	179.1>152.1(15)
Napropamide	22.54	128.1>72.0 (10)	271.2>128.1(5)
Norflurazon	26.57	303.0>145.0(20)	305.0>145.0(20)
o,p'-DDD	25.17	235.0>165.0(20)	235.0>199.0(18)
o,p'-DDE	21.45	246.0>176.0(25)	317.9>246.0(20)
o,p'-DDT	23.66	235.0>165.0(20)	235.0>199.0(18)
Oxadiazon	23.71	258.1>175.0(10)	304.1>260.1(10)
Oxadixyl	25.35	163.1>132.1(10)	233.1>146.1(10)
Oxaziclomefone	7.36	187.0>158.8(20)	189.0>160.7(20)
Oxyfluorfen	24.05	361.0>300.0 (12)	300.0>223.0(10)
p,p'-DDD	25.25	235.0>165.0(20)	235.0>199.0(18)
p,p'-DDE	23.25	246.0>176.0(25)	317.9>246.0(20)
p,p'-DDT	26.49	237.0>165.0(20)	235.0>199.0(18)
Parathion	18.25	109.0>81.0(10)	291.0>109.0(15)
Parathion-methyl	15.64	263.0>109.0(15)	233.0>124.0(15)
Penconzole	19.99	248.0>156.7(25)	159.0>122.8(20)
Pendimethalin	19.93	252.1>162.1(12)	252.1>191.1(12)
Permethrin 1	30.37	183.0>168.0(15)	183.0>153.0(15)
Permethrin 2	30.53	183.0>168.0(15)	183.0>153.0(15)
Phenthoate	20.62	274.0>125.0(7)	146.0>118.0(10)
Phorate	10.93	260.0>75.0(5)	121.0>65.0(10)
Phosalone	28.96	182.0>111.0(15)	182.0>138.0(10)

Phosmet	27.95	160.0>77.0(20)	160.0>133.0(15)
Pirimicarb	14.66	166.1>96.1(10)	166.1>137.1(10)
Pirimiphos-methyl	17.25	290.1>233.1(10)	290.1>125.0(15)
Pretilachlor	23.35	162.1>132.1(15)	262.1>202.1(15)
Probenazole	12.56	159.0>130.0(5)	159.0>103.0(15)
Procymidone	20.89	283.0>96.0(15)	285.0>257.0(10)
Profenofos	23.10	338.9>269.0(20)	336.9>188.0(30)
Prometryn	16.35	241.1>184.1(15)	226.1>184.1(12)
Propachlor	9.29	196.1>120.0(10)	176.1>120.0(10)
Propanil	15.44	217.0>161.0(10)	219.0>163.0(10)
Propargite	27.17	173.1>135.1(12)	173.1>105.1(12)
Propazine	12.37	214.1>172.1(12)	229.1>214.1(10)
Propiconazol 1	26.41	259.0>173.0(20)	261.0>175.0(20)
Propiconazol 2	26.61	259.0>173.0(20)	261.0>175.0(20)
Propyzamide	13.00	173.0>145.0 (15)	173.0>109.0(18)
Pyraclonil	30.00	299.0>256.6 (15)	279.0>262.6(25)
Pyriproxyfen	29.11	136.0>78.0(15)	136.1>96.0(15)
Quinalphos	20.58	146.0>118.0(15)	274.1>121.0(10)
Quizalofop-ethyl	31.63	372.1>299.1(15)	299.1>192.1(26)
Simetryn	15.88	213.1>170.1(10)	213.1>185.1(10)
Spirodiclofen	30.35	312.1>259.1(10)	312.1>109.0(10)
Sulfotep	10.75	322.0>146.0(25)	202.0>146.0(15)
Tebuconazole	26.92	250.1>125.06(20)	252.1>127.1(20)
Terbufos	10.93	231.1>203.0(10)	231.0>175.0(15)
Tetramethrin 1	27.97	164.1>107.1(17)	164.1>135.1(10)
Tetramethrin 2	28.16	164.1>107.1(17)	164.1>135.1(10)
Thifluzamide	24.23	194.0>166.0(12)	448.9>428.9(10)
Thiobencarb	17.62	257.1>100.0(10)	100.0>72.0(12)
Tolclofos-methyl	15.82	264.9>250.0(15)	265.0>220.0(20)

Tolfenpyrad	34.37	383.2>171.1(20)	383.1>145.1(20)
Triadimefon	29.53	208.1>181.1(10)	208.1>127.0 (10)
Triadimenol	20.62	168.0>70.0(10)	128.1>65.0(10)
Triallate	13.97	268.0>184.0(22)	270.0>186.0(22)
Triazophos	25.95	257.1>162.0 (10)	161.0>134.0(10)
Trifloxystrobin	24.25	116.0>89.0(15)	131.0>130.0(10)
Trifluralin	10.57	264.1>160.0(15)	306.1>264.1(15)
Vinclozolin	15.65	212.0>172.0(15)	285.0>212.0(15)
Zarilamid	24.67	189.0>124.9(10)	293.1>154.4(15)

Table S5.Sample collection in each city of Shandong Province

	Jinan		Taian	Weifang		Linyi	Liaocheng	Binzhou
	Zhangqiu	Xiazhuang	Feicheng	Anqiu	Shouguang	Cangshan	Guanxian	Boxing
	Market<br>Base	Vegetable base	Supermarket<br>Vegetable base	Vegetable base<br>Supermarket	Market<br>Vegetable base	Market<br>Vegetable base	Market	Supermarket<br>Vegetable base
Strawberry	2	2	2	1	2	2	1	2
Cucumber	2	1	2	2	2	2	1	2
Watermelon	2	1	2	1	2	1	1	1
Lotus root	1	1	1	1	2	1	1	1
Melon	2	1	2	1	3	1	1	1
Leek	4	2	4	2	4	4	1	4
Leaf lettuce	1	1	2	1	1	1	1	1
Crown daisy	2	1	3	2	3	2	1	2
Asparagus	1	1	1	1	1	1	1	1
